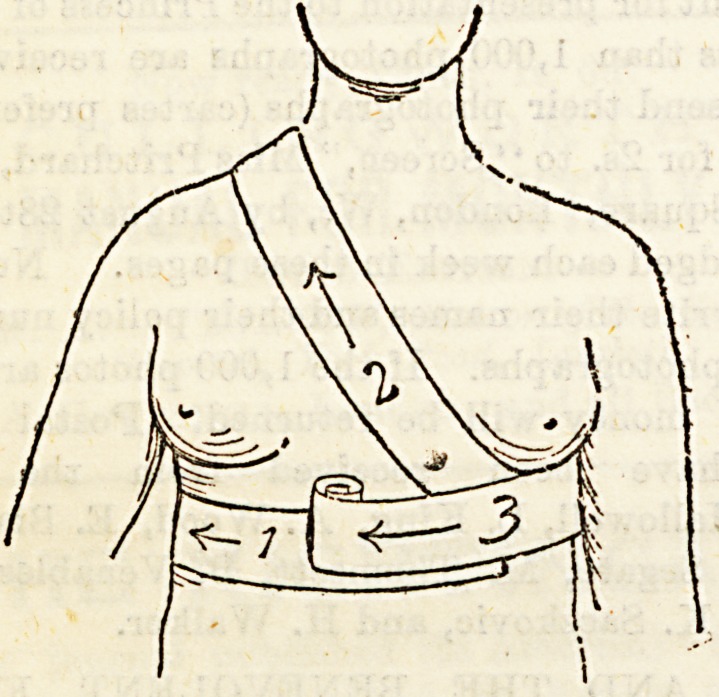# The Hospital Nursing Supplement

**Published:** 1891-08-08

**Authors:** 


### "The Hospital" Nursing Mirror.

Being the Extra Nursing Supplement of "The Hospital" Newspaper.

Contributions for this Supplement should be addressed to the Editor, The Hospital, 140, Strand, London, W.C., and should have the word "Nursing" plainly written in left-hand top corner of the envelope.

### En Passant.

OBSTETRICAL SOCIETY.—A very useful piece of work has been done by the Midwives Institute, which has published a list of the midwives holding the Diploma of the Obstetrical Society of London, from 1872 to 1891. During the first year only six nurses took the diploma, during last year 159 nurses took the diploma. All hospital matrons and secretaries should possess this list, which has been prepared and published, with the authority of the Society, and is likely to be a true means of protecting the public from ignorant and inefficient midwives. The list can be had from Mrs. Nichol, Nurses' Club, 12, Buckingham Street, Strand. Price 3d.

SISTER FRANCES.—On Monday the 27th inst., a Drawing-room meeting was held, by the kind permission of Mrs. Goslett, at Coldblon, Banstead, for the purpose of introducing Sister Frances, who spoke on behalf of the St. Luke's Home, Vancouver. The Rector, the Rev. F. Uniacke, presided, and gave a brief explanation of the subject. Sister Frances, following, gave a short account of the Home and its work. The Rev. Canon Hoste, of Farnham, endorsed much of what had been said, and spoke of the personal help rendered his son, who two years ago fell ill of typhoid fever, was nursed in the Home, restored to health, and sent back to him well. A vote of thanks to Mrs. Goslett was passed, on the motion of the Rev. C. Salmon. The donations from those present amounted to £7 11s. 2d., and a cheque for that amount was presented to Sister Frances, many promises of future help being made at the same time.

THE ROYAL RED CROSS.—All sorts of stories of women's heroism in India are now being recalled, and wonder expressed that they also did not receive the decoration lately accorded to Mrs. Grimwood. A certain amount of envy must mar every honour, we suppose. Mrs. Cawley and Mrs. Damant are said to have played a courageous part in the defence of Kohima in 1879. On one occasion, after a short respite, when Mrs. Cawley was in the lines tending the women and children, the firing began, and the path up to the house being right in view of the Naga sharpshooters, it was necessary for her to run for it. A constable voluntarily accompanied her, keeping the outer side, so that he, to some extent, protected her at the risk of his own life. Occasionally it was necessary to go to a small godown, where the stores were kept, and on one occasion a bullet came though the wall not far from Mrs. Cawley, and a second close by Mrs. Damant. On the 22nd the two ladies happened to show themselves outside the back-door, and had only just shown themselves for a moment when two bullets from the opposite hill struck the earthwork of the stockade straight in front of them, and not twenty yards off. Mrs. Cawley's remark on this incident in a letter home, "It made me feel very savage to be shot at in this way," is characteristic of her pluck. All this may be very true, but scarcely so characteristic of serviceableness and care of the wounded as it is of courage. These ladies seem to want orders of merit for having stuck to their husbands and shared their risks, and certainly this virtue is so rare it demands reward. But these faithful wives should not trespass on the field of the Nursing Sisters, who go deliberately into danger at the call of strangers, being themselves only poor spinsters. Some other order should be invented for the women who keep their marriage vows.

MINIATURE CHARTS.—We have received from H. K. Lewis, a bundle of "Small Clinical Charts." They are only about eight inches by four, and each holds memoranda of a case for a month. They are most clearly and carefully ruled, and doubtless a precise young student or neat nurse might keep them legibly, but the old-fashioned doctor with the gold-rimmed spectacles would stare aghast at such finnicking things. They ought to be specially useful in keeping a pocket record of cases, as a dozen of these charts would make no appreciable difference in the bulk of a pocket-book.

THE PRINCESS OF WALES AND THE NURSES.—It has been decided that a screen containing the photographs of the first and second thousand nurses will be the most acceptable gift for presentation to the Princess of Wales, provided not less than 1,000 photographs are received. If the nurses will send their photographs (cartes preferred) and a postal order for 2s. to "Screen," Miss Pritchard, The Lodge, Porchester Square, London, W., by August 28th, they will be acknowledged each week in these pages. Nurses are requested to write their names and their policy number on the back of the photographs. If the 1,000 photos are not forthcoming, the money will be returned. Postal orders for 2s. each have been received from the following nurses: S. Hallowell, L. King, A. Wood, E. Bishop, R. A., F. Elms, P. Segate, M. Thomsett, B. Venables, C. Dunn, S. Anthony, K. Saczkovic, and H. Walker.

NURSES AND THE BENEVOLENT FUND.—For some time past the Committee of the Benevolent Fund of the Royal National Pension Fund has been at work, under Lady Rothschild's presidency, inquiring into the applications for aid which have been made to them by nurses, and they have now dealt with all the cases which have come before them. The objects of this fund are already well-known to nurses, but they do not perhaps realize to how great an extent they can aid in extending its action so as to benefit all their fellow-workers, who from ill-health or other causes are not so well circumstanced as they are. Besides this, it is even possible, for one never knows what changes one may experience in this life, that in a measure those who aid in strengthening the fund, may be building up for themselves what may prove to be a much-needed means of help in future years. How can nurses help? In two ways. First, each of the nurses who belong to the Pension Fund might set to work to persuade some one person to subscribe ten shillings or a pound a-year to the Benevolent Fund, who will so obtain the right of nominating a nurse for relief, and they would thus be providing an excellent means of enabling it to do much more work than it is now able to perform. Some nurses, however, may say that they are quite unable to do this, their friends could not afford to do it. For such nurses there is still some work to do which will help their co-workers, and we therefore ask them the following question: What better can each nurse, who now being a member of the Fund feels perfectly secure, do than to persuade one other of her fellow nurses to become a policy-holder in the Royal National Pension Fund? We shall be glad to have any letters containing suggestions on the points we have raised, and we hope that very many of the two thousand members of the Fund will make up their mind at any rate to try to induce one other nurse to become a policy-holder forthwith. Full particulars of the many and great benefits which nurses and hospital officials will derive from joining the Fund are given in an article by Mr. Burdett, reprinted from the *Daily Graphic*, copies of which (as well as prospectuses) can be procured from the Manager of the Fund, 8, King Street, Cheapside, London, E.C.

### Lectures on Surgical Ward Work and Nursing.

By Alexander Miles, M.B. (Edin.), C.M., F.R.C.S.E.

XXXII.—BANDAGES FOR THE TRUNK.

Bandages for Mamma.—The ascending spica is again the form of bandage selected, the object being, as a rule, to give support in cases of inflammation and suppuration of the breast. Supposing the *left* mamma to be the one affected, place the tail of the bandage against that side of the chest just below the breast, carry the bandage towards the right, and go round the body. As you reach the starting point catch in the tail, and elevating the inflamed gland with the palm of the hand carry the bandage across the chest, so that it will take the place of your supporting hand. Pass over the right shoulder and across the back to the starting point, thus completing the first figure of 8. Similar turns are applied, each going higher than the one preceding it, till all the mamma is covered in and supported.

In cases where both breasts are the seat of inflammation two such bandages should be applied separately, rather than the double spica in which the pressure on one side is directed from below upwards, and on the other in the opposite direction. This—the double spica—is rarely, if ever, indicated in preference to two single bandages.

Bandage to Retain Dressing after Excision of the Breast.—As this bandage is first applied while the patient is still only semi-conscious from the anæsthetic, it will be described as she lies on her back in bed. We shall suppose the right breast to be the one removed, and that the necessary dressing has been applied and the arm flexed to a right angle and placed across the front of the body. Protect the *sound* breast by a layer of wool, especially if it be pendulous, and also the axilla, and begin the bandage by laying the tail over the sound breast, and carrying it across the dressing to pass over the right arm just below the shoulder. Pass under the back to the point at which you started and there catch in, and so fix the tail of your bandage. Another turn is made round the body at a slightly lower level than the first, and continued across the front as far as the affected elbow. At this point the direction is changed, and the bandage is made to travel across the back to the left shoulder, in doing so supporting the right elbow. It then goes obliquely across the front of the body to the right elbow, round the tip of it, and thence up along the back of that arm to the shoulder, after crossing which it runs across the chest obliquely from right to left, thus making a St. Andrew's Cross with the previously made oblique turn. Passing to the back, and there also making a St. Andrew's Cross by running to the right shoulder, a turn is carried down the *front* of the affected arm, corresponding to a similar turn already made along the back of it. Looping round the elbow once more, a turn goes across the back to the left shoulder, and thence vertically down to the lower margin of the dressing on that side, where the bandage ceases. Pins are inserted liberally at all the points of intersection, and thus is secured a very efficient mammary bandage. Such a bandage may, of course, be used also for any other condition in which it is necessary to fix the upper extremity.

*(To be continued.)*

### Examination Questions.

The following questions on Masso-Therapeutics, set at the Grafton College examination, may be interesting to such of our readers as are masseuses :—

1. Describe in full the methods of central galvanisation.
2. To what parts of the body should the hand or hands of the operator be used as electrodes when coil currents are employed, and why ?
3. What are the objections to administering an *electric bath* with both electrodes in the water ? Describe any methods you think preferable.
4. Define the massage movements known as "muscle rolling" and "muscle vibrating."
5. Name the *percussion* movements and state the effects you hope to produce by their use upon the parts treated.
6. How would you proceed to massage a patient suffering from constipation ? State clearly the parts to which you would give greatest attention and name the movements you would employ.
7. Name the *exercises* used in treatment of a stiff shoulder joint, stating also the position the patient should be placed in.

### Christmas Competitions.

We want none of our readers to go away for their holidays without taking some piece of work for wet days, which, when finished, can be sent to us for our Christmas parcels. So heartily were the garments for adults which we distributed last year appreciated, that we want this year to have twice the number. To encourage all to help us in this way, and to add interest to the work, we offer the following prizes, which will be awarded in books or money as the winners choose : (1) For the best pair of socks knitted by a nurse, 5s.; (2) for the best pair of socks knitted by any Hospital reader, 5s. ; (3) for the best made flannel shirt, 10s. ; (4) for the best made woman's blouse, 10s. ; (5) for the best made flannel petticoat, 10s. ; (6) for the best made and best shaped dressing gown for an invalid cut out and made by a nurse, 20s. It will be seen that No. 1 and 6 are reserved for nurses only. With regard to No. 6 we specially hope for many entries, and if we secure them we propose to give more than one prize. Flannellette is cheap, and light, and warm, and would, therefore, form the best material for the dressing gown. In judging, four marks are given for workmanship, four for shape, and two for general appearance ; therefore, it is not wise to spend time on elaborate trimmings. Long seams may be done by machine.

### Appointments.

British Seamen's Hospital.—On July 1st Miss Emily F. Campbell was promoted to be Matron of this hospital at Constantinople, in place of Mrs. Reilly, who resigns after thirty-four years' service. Nurse Mary Henderson Fraser, who was five years at Glasgow and three in Edinburgh Royal, takes Miss Campbell's vacated post of chief nurse. We wish to both every success.

Ayr County Hospital.—Miss Wingfield, of the Cripples' Home, Bray, has been appointed Matron of this hospital, in place of Miss Thorn, resigned. Miss Wingfield trained at Addenbrookes.

Miss Mary Laing, staff-Nurse in the Royal Infirmary, has been appointed to the new office of Superintendent of Nurses in the Glasgow Town's Hospital ; and Staff-Nurse Christina Robertson has been appointed Matron to the Glasgow Samaritan Hospital.

### Reading for the Sick.

### GOD'S TAPESTRY.

There was a curious kind of needle-work, called tapestry, used in old times for wall hangings, and which is being revived now for the same purpose in halls and churches. The stitches in it are innumerable, and the shades of colour so varied as to give, when finished, the effect of a lovely picture.

A very beautiful example has lately been put up at the south-east end of the chapel of Exeter College, Oxford, which was designed by one of our best modern painters for a large space of wall requiring decoration. The subject is the leading by a star of the Magi, who have travelled from a far country to seek Christ, and who are bringing rich offerings to present to the infant Saviour. The central figure, supposed to be invisible to the Wise Men, is an angel of large and majestic proportions, holding in his hands the star which has guided them on their way. From the right the Magi approach with reverence towards the stable in which the Holy Family has found shelter. The devotion of the Magi, the placid happiness of the Virgin Mother as she gazes with rapture on her Divine Son, who reposes on her lap, the watchfulness of Joseph, and the loving care of the Angel, are depicted with infinite care. The embroiderers have faithfully reproduced all these beautiful and subtle effects, and when we are told that it required ten thousand different hues and shades of worsted to produce them, the observer is filled with admiration for the patience, skill, and ingenuity which has wrought such a piece of perfection.

But the great marvel is that the work is all done on the wrong side. Each worker, and there must have been many employed, does his or her part from the back in faith and obedience. They are told they are making a beautiful picture, but to their eyes nothing but a mass of blurred colours and tangled ends presents itself. No doubt they long to know how the stitches they set look in front, and why they put in now a green, now a blue, and now a red one. But they must wait for that satisfaction till the end. Careless workers may think it matters little if they depart from the pattern and leave a few mistakes which they know they have made, contrary to their written orders. But the master who directs all can see the front, and has them altered immediately, lest his picture should be spoiled.

Our life on earth may not unaptly be compared to a piece of tapestry. God has designed a most perfect and magnificent work which we, His creatures, can neither see nor understand in full. To us it seems all tangles and troubles and worries. We cannot understand why the trials of sickness and disappointment are sent to us, and what good we are doing by bearing them patiently. Is it not because we are on the wrong side of the tapestry and so fail to see the necessity and fitness of the trials. If we do not work by our Master's laws, but strive to perform our tasks in our own way, we often put in stitches of our own devising, and are angry and discontented when they are mercifully corrected.

Wise men know the Almighty has a great and wonderful design which He wishes us to carry out, and they are content to wait until, this life ended, they cease from their labours and see it face to face. We shall be but foolish, careless labourers if we do not go on steadily and patiently from stitch to stich, doing our daily task cheerfully, bravely, bearing and forbearing, loving God with all our hearts, and asking Him to guide our hands aright that we may not mar His noble work. Now we see in a glass darkly, but if we wait patiently we shall see Him as He is. Our loving Saviour will help and solace us in all our toils and uncertainties, and will give us here that peace of mind which passeth all understanding, and in eternity we shall know all we have longed to know, and with the Blessed in Christ will sing for joy at the completion of a glorious work in which, though feebly, we have been allowed to take part.

### Wants and Workers.

*Clothing Wanted.*—Nurse Winter, Stansted, Essex, appeals for print dresses, aprons, and underclothing, or money to get the same, to send a girl out to service. Nurse Winter will gladly answer all inquiries about the case, which is a sad one.

*Acknowledgments.*—Sister Frances acknowledges £2 from raffle of a doll and a large parcel of beautifully made clothing from the servants at Coldblow.

### More Revelations about Registration.

The evidence of Dr. Bedford Fenwick before the Lords' Committee deserves more comment than we had room for at the time it was given; at least, it deserves wider publicity amongst those who do not understand the dangers and difficulties of registration; comment would be superfluous after the very searching and poignant questions put by some of the Lords. The following are a few of the questions and answers asked and given on July 18th :—

By the Earl of Kimberley: I see, looking at your list, that a great many of the nurses are not certificated; it is stated so?—It is distinctly stated so.

Now I take, quite by accident, one case of a nurse, and against her name it says "London Hospital, 1884-86; Poplar Hospital in 1886." She might have been only a month in Poplar Hospital?—That is the one thing that we wish to be most accurate about. If a nurse was only eleven months in a hospital, we should say that she had been there "in" that year.

But that is not expressed on the register. I could not have known that from reading this register; you know that I should not have known it; but in this particular case (which I took quite by accident) all I see is that the nurse was registered on such a date, and then against her name I see "London Hospital, 1884-86; Poplar Hospital in 1886." Now that does not give one the means of knowing how long she was trained in either hospital?—Poplar Hospital is not a training hospital; it is a hospital for accident. I do not know what case your Lordship is referring to.

It is the eleventh name from the beginning?—That is the form which is filled up (handing a form to Lord Kimberley).

You see my point. My point is this: I am a person inspecting the register; I see this nurse's name; in the first place I could not possibly know, being a person unconnected with hospitals, that Poplar Hospital is not a training institution; I should suppose it was one. Again, with regard to the London Hospital, I should only know that she was trained from 1884 to 1886; she might only have been 13 months there, might she not?—Exactly.

That is not definite information?—That is definite information, that she was training in the London Hospital in those years.

But that does not tell me how long?—There is an immense variety in the different hospitals in England in the length of time that is given to the training of their nurses.

But it is quite evident that the nurse did not pass her full time at the London Hospital?—Yes.

And therefore, knowing what I know about hospitals, should view that case with suspicion; I should say tha that nurse might have been dismissed from that hospita l there is no information in the register to tell me as to that—No.

And the inference I should draw, from what I know of the circumstances, would be this: unless I had further information about that nurse, I should doubt her competence?—Yes.

But would the public generally draw such an inference as I, from the knowledge which I have derived in this Committee, should draw?—Every nurse has to produce evidence of character, and that evidence of character and evidence of hospital training is carefully enquired into before she is put on the register.

In the case of the person mentioned just now who was discharged from the London Hospital, and whose name is entered upon your register, so far as that entry is concerned, it is not thoroughly trustworthy?—We have not been able to obtain information from the London Hospital as to the character of the person; but we have in every case obtained information that a nurse has been at the hospital for such a time before we have stated that fact on the register.

Still, a nurse may have been at the hospital, and may have been trained there, and yet she may have proved herself very incompetent?—Yes.

Does your register give any means to one of knowing that?—Except that we have to enquire into the character of every nurse before she is enrolled.

That is proof of her moral character, is it not? But you have no means of knowing whether she proved herself to be a satisfactory nurse unless she had a certificate?—I have said that some hospitals do not give certificates, and that is a very difficult thing to deal with.

So that there again your register is very imperfect in its information; for example, any person reading this list would naturally infer that the certificated nurses were those that had really proved themselves efficient in training; but it appears from your statement that there are other hospitals which do not give certificates, and therefore there might be nurses perfectly competent to nurse, and yet they would not appear with certificates against their names?—Yes.

Is not that an unsatisfactory result?—We think that that is one great reason for a register, to bring about some uniformity. That really proves our case, the absolute lack of uniformity, not only in training, but in guarantee to the public that nurses are trained.

Do you undertake from time to time to ascertain that these nurses continue to be of good character and efficient; because, for example, a nurse might be registered in the year 1891, and in the year 1901 she would be still upon your register; and unless you had made enquiries, and found that she was still efficient and trustworthy, she might be a most inefficient and untrustworthy person?—We hope that before 1901 the register will have been taken up by the State; but we have no means of keeping in touch with our nurses; we must accept them, and we register them on their technical training and on the evidence we have received that they are of good character when they are registered.

I understand that the real object of this registration is to bring about a close corporation of nurses?—I think not quite that. We want to protect the public against incompetent and ignorant women now practising as nurses.

Earl Cathcart then put a few questions, amongst them the following:—

Suppose I wrote to you to tell you that nurse so-and-so had been drunk on a certain occasion on duty, would a communication of that sort be actionable?—We should ask you to be good enough to verify your words.

What verification would you ask for?—We should ask you to be good enough to give us the opportunity of acting legally upon it.

Then it would render me liable to an action if the thing were not exactly proved?—We should take it for granted that you would be able to prove your statement.

Then I might say that she was drunk, meaning that she was "military drunk," that is to say, so far drunk as not to be fit for duty, and a certain other person might say that she was not drunk because she was not under the bed?—That is the difficulty; but we should hope the public would, for its own sake, tell us the facts and be willing to support their statements.

I am not mentioning now that which is hypothetical, but that which is a matter of fact. It is a difficult thing to prove that a nurse was drunk, though, as a fact, she was unfit for duty, and very likely snoring in a chair.

Lord Thring then took up the ball.

I should like to ask you one or two questions on the subject of registration. Of course everybody can see at once that the objects of your Association are most desirable, and if your rules carried them into effect they would be most efficient. Now you put down in your register that a nurse has been in a hospital for a particular time, I do not care what the time is, but for a particular time; an outsider, that is to say, a person who saw but one case of that sort, would imagine that that would be a guarantee that she was trained. Now it may be exactly the contrary; it may be that she has gone to the hospital and she has either been discharged or has left that hospital under a proof either that she is not competent or that she is not a good nurse, or not a good temper, or fifty other things. Hence, the fact of her having been in the hospital is no guarantee at all that she is a good nurse, and unless you have got a certificate it is absolutely misleading, or may be so, to put her on the register?—I think not; I think we simply state that she was in a hospital where technical instruction is given for a certain length of time.

But follow my question: A person who is not accustomed to the question of nurses, would imagine that the fact of her being in a hospital meant that she was being trained in the hospital, would they not?—So it does.

Suppose that, instead of her having benefitted by her training, she has shown herself in that hospital to be utterly unfitted to be a nurse; surely the fact of her having been in that hospital is no guarantee of her having been trained?—But it would be impossible for any body, even a legal body, to so interfere with every hospital as to say, "We do not think that this nurse is sufficiently trained."

This is not my point; but you will admit that it is still misleading in fact?—I cannot see it; it may be my own fault.

I ask you, as it stands, whether taking an ordinary person, the statement that a nurse has been trained in a hospital does not lead that ordinary person to think that she has been really trained?—I cannot say that it does, because there are no two women alike. Some women will learn very much more than others in a given time.

Supposing a servant came to you and said that she had been trained to clean plates, would you not draw the inference that she had been trained to clean them well?—If I had a guarantee from somebody who had seen her clean plates.

Here we will leave Dr. Bedford Fenwick; he exactly states our point all along, that only those who have seen nurses at their work should grant them certificates—that is a terrible admission, "*If I had a guarantee from somebody who had seen her clean plates.*" To this point, and to the point brought out by Lord Cathcart, that the Association cannot protect those who would help to weed out the bad nurses, from actions for libel, are sufficient evidence of the uselessness of the register. There is a third point we should have liked to have seen brought forward by the Lords, and that is, that as the Association is opposed by nurse-training authorities, its efforts are subversive of discipline and calculated to raise ill-feeling between the nurses and the schools. No great evil has been done yet, because so few trained nurses (in the ordinary use of the word "trained") have gone on the register; but Lord Sandhurst, as a soldier, ought to know the difficulties and dangers likely to arise if the nursing ranks are taught by an outside body to ignore their officers and the authority of their training schools. The spirit of loyalty would be utterly ruined in the nursing world if the Association was allowed to have its own way; and this is a fact the Royal President of the Association should have brought to her notice.

### The Princess of Wales' Fund for Mrs. Grimwood.

The following sums are acknowledged: Nurses Blayney and L. C. Garling, and a Matron, 5s. each; Nurse Doran Mackay and friends, 4s.; An Army Nursing Sister from Malta, Nurses Spreng and "W.", 2s. 6d. each; One of the Second Thousand, No. 537 Pension Fund, Nurses S. A. Clark and Cook, 2s. each; Nurses Moorhead, Pennington, Puxley, Sophia Hallowell, Grey, Reeve, Eugénie Merme, E. H. Exmouth, F. Stockwell, and One of the Second Thousand (Clifton Institution), 1s. each. The list is now closed.

Erratum.—The name of Nurse C. E. Parker was erroneously given as Parkes last week.

### Presentation.

On Friday, the 31st ult., a purse and fifteen sovereigns was presented to Staff Nurse Margaret Aitken, on the occasion of her retiring after having completed a period of twenty-five years' service as a nurse in the Glasgow Royal Infirmary. The presentation was made in feeling terms, and in the name of the nurses, by Mr. William M'Ewen, who was Chairman of the House Committee at the time Nurse Aitken was appointed, and through whose efforts the nursing was brought to its present high state of efficiency. His successor, Mr. Hugh Brown, who continues the good work, was also present, and spoke warmly of the way in which Nurse Aitken had discharged her duties.

### Everybody's Opinion.

### WORK IN VANCOUVER.

SISTER FRANCES writes: Will you allow me before leaving England to thank the nurses who so generously responded to my appeal. I have received many letters of sympathy and promises of help. I have been four months at home, and return to Canada refreshed and ready to begin my work with hosts of new friends, heaps of proofs of their willingness to help, invitations from institutions, presents of money, and many useful gifts for us all, and great will be the rejoicing when I open my packing case. And now a word upon a subject that gave me a glimpse of nurses and their readiness to welcome a stranger. I was present, as many who read this will know, on Saturday at Marlborough House, and very proud I felt at my reception; it was to me a most unexpected reward. I was only for about a moment alone, when two nurses ran up exclaiming, "Here she is; we must not let you feel a stranger; please let us go about with you," and so for about an hour one after another came up to speak, and I was so truly pleased to meet two of my own Guild Sisters. And so in this, my last few days in England, I can look back and speak of when abroad, not only my introduction to the Princess of Wales, but of a pleasant and happy day spent among several hundred fellow nurses in England, and I wish very specially to thank the young infirmary nurse who spoke to me when leaving, and asked me to accept her donation to the Home. I sail in the "Parisian." Good-bye!

### THANKS TO ALL.

"J. R. D." writes: At the Princess of Wales' reception on Saturday many of the nurses desired to express their thanks to every one who by their kind thought contributed to the enjoyment of the nurses. As it could not be done individually, we agreed to ask you to do it for us through THE HOSPITAL, for, added to the honour of being received by our dear Princess, there was thorough enjoyment. So good and self-sacrificing it was of the Princess to give the certificates with her own hands, when she was so far from well. To Lady Cadogan, too, our best thanks are offered for the delightful evening we spent at the Merchant Taylors' Hall, and to al who so kindly undertook the entertainment.

### A PROTEST.

K. M. HEANLEY, Boston Hospital, Lincolnshire, writes: As your paper has a large circulation amongst nurses, will you help me to make known to members of R.B.N.A. that there are many of us who object to the article in *Nursing Record*, entitled "Great is Bumbledom," published apparently in our interests; also to make known that the *Nursing Record* is not the official organ of the R.B.N.A.? I had intended to make these two points clear at Lincoln, July 30th, but the editor of *N.R.* frightened our Hon. Secretary, and through him most of the members present, by threatening legal proceedings if I brought forward a resolution on these points. I, therefore, told our Hon. Secretary that as I could not speak at what should be a privileged meeting, I should lay the matter before the medical press. He asked why I did not bring forward a resolution against THE HOSPITAL for writing against us in the strong terms which you do. I am, however, not concerned in objecting to an openly expressed disapproval (which any member may meet as he or she thinks fit), but with the character of articles which, while appearing to favour us, do us really a great deal of harm in exciting the strong disapprobation of those whom we should wish to interest in the true and laudable objects of our society.

### REGISTRATION.

"FAIRPLAY" writes: The article in last weeks' issue on the B.N.A., seems to me rather misleading concerning the trained monthly nurses.

Its register is decidedly at fault if it classes a nurse who has gone only through midwifery nursing as a fully-trained nurse. The safest midwifery nurses are those who stick closely and exclusively to their own branch of the profession and keep clear of surgical operations and infectious cases outside the pale of their own branch.

The days of "Sairy Gamp" are over as well as of "Betsy Prig," and the trained, intelligent, midwifery nurse of to-day, who gains her knowledge at such institutions as Queen Charlotte's Hospital in London, or indeed, at any other well-managed lying-in hospital, is capable of coming very well to the front in her own special line. The writer of the article last week says, "The public may have untrained help if it likes." The expression is not quite kind. The fault lies with the register, and also with the nurses who place themselves, or are placed upon it. They should be above placing themselves in a false position, and remember that a Jack-of-all-trades is not unfrequently master of none! I do not write for the sake of controversy, but in a spirit of fairness to those worthy women who are conscientious enough to go quietly on in their own line, and into whose keeping the welfare of those important members of society, the wives and mothers of our land, is entrusted at the most critical period of their existence.

### LODGINGS IN LONDON.

"A PROVINCIAL MATRON" writes: As one of the second thousand who enjoyed the free home-like comfort of the "Nurses' Hotel," 18, Royal Avenue, Chelsea, combined with the friendly care of the Lady Superintendent, I would highly commend it to all nurses wishing to spend either a short or lengthened holiday in London. Charges are moderate, and while being conveniently situated, a comparative quietness from the busy thoroughfares is enjoyed, which adds not a ittle to its other attractions.

### Notes and Queries.

### Queries.

(29) *Address wanted* of a convalescent home about twenty miles from Liverpool; it is some distance from the railway. Some of the patients pay, some are free. I think the first syllable is "Wood."—*W.*

### Answers.

*Mary B.* must send name and address.

*L. T.*—Apply at any hospital, no one will stop you.

*G. M. E.*—A Queen's nurse is a district nurse working in connection with the Queen Victoria Jubilee Institute. The only advantage is the name and the badge.

*Private Nurse.*—Read any good book on midwifery. You have to supply the Obstetrical Society with proof that you have attended a lying-in hospital for not less than three months, and have personally attended 25 labours.

*Babies' Ears.*—Miss Adelaide Claxton's apparatus, price 3s. 6d., from 62, Strand, W.C. It does not hurt the child's ears, and keeps them in position.

*Nurse Mary.*—The Levick Institution, 34, Rue de Prony, Parc Monceau, Paris.

*Anonymous.*—We do not prescribe.

(25) There is no need to empty a water bed, no matter how long it is in use. All that is necessary is to take out a couple of large cans of water and put in the same quantity of warm water about every third day in winter and once a week in summer. This will keep the bed an even warmth. I have a patient who has been more than a year on a water bed, and except for a change of rooms the bed has not been emptied. I am afraid nurse will find the water rather smelly after being left undisturbed for three months, but if she changes it in the way I have described *every day* for about a week it will get all right. I have found it best to empty the water at one end of the bed and pour fresh in at the other.

*J. R. D.*—Thanks for your letter. Unfortunately we cannot print it all.

*Medicus.*—You will find the terms and information you require fully stated in *The Hospital Annual*, 140, Strand, W.C., Price 2s. 6d. Each convalescent institution has its own terms for admission.

### Amusements and Relaxation.

**SPECIAL NOTICE TO CORRESPONDENTS.**

**Third Quarterly Word Competition commenced July 4th, 1891, ends September 26th, 1891.**

Competitors can enter for *all* quarterly competitions, but no competitor can take more than *one first prize* or *two* prizes of any kind during the year.

The word for dissection for this, the SIXTH week of the quarter, being

"THUNDER."

| Names. | July 30th. | Totals. | Names. | July 30th. | Totals. |
|---|---|---|---|---|---|
| Paignton | 83 | 178 | Christie | — | 44 |
| Psyche | 78 | 176 | Dulcamara | 80 | 170 |
| Hope | — | 47 | Nurse J. S. | 84 | 177 |
| Lightowlers | 90 | 187 | Qu'appelle | — | — |
| Wizard | 60 | 151 | E. M. S. | — | 68 |
| Wyameris | — | 46 | Jenny Wren | — | — |
| Dove | — | 62 | Carpe-diem | — | 65 |
| Punch | 88 | 179 | Grannie | — | 36 |
| Ivanhoe | 74 | 168 | Nurse G. P. | 44 | 123 |
| Tinie | — | — | Goodnight | 49 | 101 |
| Agamemnon | 89 | 184 | Gamp | 55 | 86 |
| Nurse Ellen | — | — | Charity | 41 | 64 |

All letters referring to this page which do not arrive at **140, Strand, London, W.C.**, by the *first post on Thursdays*, and are not addressed **PRIZE EDITOR**, will in future be disqualified and disregarded.

### Royal British Nurses' Association.

### EXTRAORDINARY TACTICS.

The third annual meeting of the Royal British Nurses' Association was held at the Science and Art Schools, Lincoln, on July 30th. The chair was occupied by Dr. Mitchenson (Lincoln).

### Formal Business.

On the motion of Dr. Bedford Fenwick, seconded by Miss Beechcroft, the annual and financial reports were adopted. They showed that the year had commenced with 3,024 members, 135 of whom had resigned; that 24 new members had been admitted, and that the total membership at the present time was 2,865. The receipts, including £158 17s. 7d. as balance brought forward last year, amounted to £1,004 0s. 4d., and the expenditure to £883 6s. 7d., leaving a balance in hand of £120 13s. 9d. The Journal account showed a favourable balance of 14s. 9d.

Two badges, one of silver for the members of the General Council, and the other of bronze for the members of the Association, were handed round for inspection, and will be brought up for adoption at the meeting of the General Council.

A provisional list of members for the ensuing year's General Council was accepted, it being stated, however, that some of those included had not consented to serve.

Certain of the bye-laws were altered, and Dr. Bedford Fenwick gave notice that at the next general meeting a resolution shall be proposed, carrying out the resolution passed at the last General Council: "That on and after January 1st, 1892, only registered nurses and midwives shall be eligible for membership of the Association."

### The "Nursing Record."

The next business was to receive a resolution by Miss K. N. Heanley (Matron of the Boston Hospital) *re* this publication.

Dr. Fenwick said: With reference to that, before anything is done, I have to read a letter which I have received—a very serious letter—from Willett and Lovegrove, solicitors. "Dear Sir,—I am instructed by my client, the editor of the *Nursing Record*, to inform you that he has received from Miss Heanley notice of her intention of submitting the following resolution"—[here follow words of the resolution left unread.]—" and to give you notice that if such resolution be submitted, my client will be reluctantly compelled to take proceedings against your Association, similar to those I am about to commence against Miss Heanley." The resolution refers, I may say, to the tone of the *Nursing Record*, and I am informed by a lawyer that those present, if this resolution is submitted to the meeting and carried—those present are also liable for endorsing her views, even if they only vote for it. That, of course, is a very serious position for the Association. It is a very serious position for us. I myself strongly object to be dragged into a law-suit and to have the expense of it, and I myself should not vote. The only question is whether it is wise for us, in the face of this letter, to proceed in the matter.

Miss Heanley: Dr. Fenwick, I may state this, that I have consulted an experienced editor, and he tells me it is quite unnecessary that I should have sent that resolution to the *Nursing Record*. Of course, taking your advice, I did so, and it was the more straightforward and honourable course, and, of course, it was one that I wished to follow, but certainly it must be a most unusual thing for any editor to threaten either an association or an individual with a resolution which has not been yet made public. Further, if you read a private letter at this meeting, surely it must be a privileged meeting, and I surely should, therefore, be able to stick to my resolution. I cannot see that it any way draws in the whole number of the Association, unless they choose to back me up themselves individually. I consulted my lawyer upon this point myself, because I also received a lawyer's letter, and another threatening me through my committee. He thinks the editor went out of his way to try and induce my committee by letter to them, to prevent my action, and surely that was scarcely gentlemanly, if it was legal. That is all. Of course, the whole of the Association is not drawn in by that. He did not say that any words I used were actionable, but if they were I should certainly leave out those words which the editor of the *Nursing Record* considered defamatory. That is legal advice upon the subject. It appears to me such a remarkably unusual thing that any editor should go out of his way to send a notice before the meeting before it was actually brought before us, when I had gone, perhaps, out of my way, as it appears, to do the civil course by him, and give him a notice, which I need never have done. The only notice that need be given on these occasions is when the organ happens to be *the* organ of the association, which the *Nursing Record* is not, and I am most anxious at this meeting we should fully understand that it is not, and we should protest against its tone for that reason. But I consider myself, therefore, perfectly permitted to bring forward this resolution.

Dr. Fenwick: I did tell you that I thought it was the only honourable and straightforward course, as you have said.

Miss Heanley: I took it as such, and I did it as such, only it is not the usual course, and you see what it has brought upon me—that course. It is certainly a most unusual course that any editor should threaten me, and threaten me at a meeting and through my committee, with legal proceedings on the resolution which has not come before you, except, as you say, in a private way.

Dr. Fenwick: I do not think, if you will excuse me saying so, that you could object to the editor of the *Nursing Record* defending himself. I, as you know, wrote you the moment I got a copy of the resolution, and acknowledged it, and it was placed on the agenda paper. There the matter stood. Then the letter stated we shall find ourselves landed in legal proceedings, and I think we are most thankful to you, Miss Heanley, for giving the editor that straightforward notice first of all, to enable him to defend himself. I was going to say it is a matter which has absolutely nothing to do with this Association. We are a very powerful and a very successful one, and we have a right to our private opinions, and we have a right to express them publicly, but we have no right to drag in a whole association of people to express opinions about a public paper which might land the Association in legal proceedings. That seems to me the only course.

Miss Heanley: They cannot be dragged in unless they vote as individual members. That is my contention.

The Chairman: I understand if this resolution were passed, it would make this meeting responsible for this action.

Miss Heanley: That is what I contend it will not do.

The Chairman: In that case, what would be the object of passing the resolution?

Miss Heanley: Simply because I wish the members to help me. It is the only way of bringing things forward, in withstanding this tone which is permeating the ranks of nursing society, and that the *Nursing Record*, which appears to the minds of us who have no means of judging otherwise, to be intimately associated with our society, and to bring forward such articles in apparent defence of us.

The Chairman: It is a most serious matter, and I think the great thing is in this way. There is a doubt whether or not you would make yourselves responsible for this, and I think it is my duty to put it to you whether, under these circumstances, this resolution shall be proposed or brought before you.

Dr. Fenwick: I may also point out Miss Heanley has a perfect right to write to the editor of the *Nursing Record* and to complain, without dragging us into it.

Miss Heanley: Have not I a right to bring it before this meeting? How can we, as members, otherwise represent our opinions? I have a perfect right that the meeting shall decide.

The Chairman: There being some doubt as to whether or not this resolution might not involve us in legal difficulties, I will ask whether it is your pleasure that this resolution shall or shall not be brought before you to-day.

Mr. Cant proposed that the resolution be not submitted.

Dr. Simpson seconded.

Miss J. Browne proposed, as an amendment, that the resolution be submitted.

Miss A. Beadwell (Matron of the Louth Hospital) seconded, and, on being put to the meeting, two supported.

The original motion was then put and carried.

This concluded the business.

Votes of thanks to various persons brought the proceedings to a close.

## Figures and Tables

**Figure f1:**